# The Emerging Role of Curcumin in the Modulation of TLR-4 Signaling Pathway: Focus on Neuroprotective and Anti-Rheumatic Properties

**DOI:** 10.3390/ijms21072299

**Published:** 2020-03-26

**Authors:** Maria Antonietta Panaro, Addolorata Corrado, Tarek Benameur, Cantatore Francesco Paolo, Daniela Cici, Chiara Porro

**Affiliations:** 1 Department of Biosciences, Biotechnologies and Biopharmaceutics, University of Bari, 70125 Bari, Italy; mariaantonietta.panaro@uniba.it; 2 Rheumatology Clinic, Department of Medical and Surgical Sciences, University of Foggia, 71121 Foggia, Italy; ada.corrado@unifg.it (A.C.); francescopaolo.cantatore@unifg.it (C.F.P.); daniela.cici@gmail.com (D.C.); 3 College of Medicine, Department of Biomedical Sciences, King Faisal University, Al-Ahsa 31982, Saudi Arabia; tbenameur@kfu.edu.sa; 4 Department of Clinical and Experimental Medicine, University of Foggia, 71121 Foggia, Italy

**Keywords:** Curcumin, TLR-4, Neuroinflammatory diseases, Rheumatic Diseases, miRNA, exosomes

## Abstract

Natural products have been used in medicine for thousands of years. Given their potential health benefits, they have gained significant popularity in recent times. The administration of phytochemicals existed shown to regulate differential gene expression and modulate various cellular pathways implicated in cell protection. Curcumin is a natural dietary polyphenol extracted from *Curcuma Longa Linn* with different biological and pharmacological effects. One of the important targets of curcumin is Toll-like receptor-4 (TLR-4), the receptor which plays a key role in the modulation of the immune responses and the stimulation of inflammatory chemokines and cytokines production. Different studies have demonstrated that curcumin attenuates inflammatory response via TLR-4 acting directly on receptor, or by its downstream pathway. Curcumin bioavailability is low, so the use of exosomes, as nano drug delivery, could improve the efficacy of curcumin in inflammatory diseases. The focus of this review is to explore the therapeutic effect of curcumin interacting with TLR-4 receptor and how this modulation could improve the prognosis of neuroinflammatory and rheumatic diseases.

## 1. Introduction

Numerous studies have reported therapeutic benefits of plant-derived bioactive compounds and their increasing use as monotherapy or as an adjunct therapy along with conventional medicines in the treatment of various diseases.

In this respect, phytochemicals have reported to exhibit a variety of biological effects on cells, mitigating necrotic cell death, modulating cellular antioxidant defense systems, reducing oxidative stress, restoring the redox balance, and subsequently protecting cells against mitochondrial dysfunction and attenuates inflammation. Therefore, they are used as multiple health-promoting or disease-preventing compounds [1].

Curcumin (CUR), a bioactive phytochemical, is a yellow pigment discovered in 1815 by Vogel and Pelletier [2] and has been consumed for >2000 years in Asian countries, for its efficacy in the control or treatment of a large number of inflammatory diseases.

Indeed, CUR was used in traditional medicine for the treatment of biliary and hepatic disorders, cough, diabetic ulcers, rheumatism, and sinusitis [3].

The molecular basis of CUR attributed anti-inflammatory effects in modulating the immune system responses has been the object of over 10,000 studies in the last two decades.

Inflammation is an adaptive physiological response induced by deleterious circumstances including infection and tissue injuries. Inflammation can be divided into two main stages: acute and chronic. The initial stage of inflammation (acute inflammation) induces the activation of the immune system, which persists only for a short time and is usually beneficial for the host. When the inflammation lasts for a longer time, the second stage of inflammation (chronic inflammation) starts and may initialize various chronic diseases such as obesity, diabetes, arthritis, pancreatitis, cardiovascular, neurodegenerative and metabolic diseases, as well as certain types of cancer [4].

Consequently, acute inflammation is beneficial, whereas chronic inflammation represents a source for several chronic diseases. In this regard, increasing evidence indicates that long-term chronic inflammation mediates several chronic diseases including arthritis and neurological diseases.

Curcumin has a wide range of therapeutic benefits and exerts anti-inflammatory effects through several signalling pathways that are associated with inflammation including Toll-like receptor-4 (TLR-4) pathway.

TLR-4 is an innate immune receptor present on the surface of immune cells and plays an important role in innate and adaptive immunity by generating a large number of cytokines and proinflammatory cytokines via the MyD88-dependent (MyD88)-dependent and MyD88-independent pathways [5].

TLR-4 signaling pathway is associated with inflammatory response and administration of CUR, could inhibit TLR-4 activation and its downstream pathway.

Taken together, the aim of the present review is to describe how CUR, with its potential anti-inflammatory effects, is able to modulate TLR-4 signaling pathway in neuroinflammatory and rheumatic diseases.

## 2. Curcumin

The use of nutraceuticals, dietary supplements, and functional foods has been steadily gaining popularity due to the increased interest in natural products and their potential health benefits [6,7].

CUR (diferuloylmethane) is the principal Curcuminoid in turmeric, the Indian spice derived from the plant *Curcuma longa Linn* (family *Zingiberaceae*), and it is commonly used in the Asian continent, especially in India.

The IUPAC (International Union of Pure and Applied Chemistry) name of CUR is (1E,6E)-1,7-bis (4-hydroxy-3-methoxyphenyl)-1,6-heptadiene-3,5-dione, with a chemical formula of C_21_H_20_O_6_ and a molecular weight of 368.38 g/mol. The chemistry of CUR is at the basis of its several biological activities. Indeed, its pharmacological effects are exerted by several functional moieties including phenolic hydroxyl groups, the central bis-α, β-unsaturated βdiketone, double-conjugated bonds, and methoxy groups [8].

CUR is hydrophobic as well as lipophilic, it has poor solubility in water or hydrophilic solutions, while it is highly soluble in organic solvents including methanol, ethanol, acetone and dimethyl sulfoxide [9]. It absorbs light, with a maximum wavelength of approximately 420 nm, which is what gives turmeric its yellow color [10].

The curcuminoid complex, found in the rhizome of turmeric (2.5–6%), contains CUR, demethoxycurcumin, and bis-demethoxycurcumin [11].

Polyphenols such as resveratrol and CUR, as well as flavonoids, were considered as the plant’s defensive response against stress from ultraviolet radiation, pathogens, and physical damage. Resveratrol and CUR are anti-inflammatory, cyto- and DNA-protective, anti-diabetic, anti-cancer, and anti-aging dietary compounds [12,13,14,15,16]. These properties have been supported by several in vitro and in vivo studies and clinical trials [17,18,19].

The hydrophobic nature of CUR is responsible for its low water solubility and the rapid intestinal/hepatic metabolism limits its oral bioavailability, impeding clinical development of curcumin as a potential therapeutic agent [20].

The US Food and Drug Administration (FDA) have approved Curcuminoids as “Generally Recognized As Safe” (GRAS) [13], good tolerability and safety profiles have been shown by clinical trials, even at doses between 4000 and 8000 mg/day [21] and at doses up to 12,000 mg/day of 95% concentration of three curcuminoids: curcumin, bisdemethoxycurcumin, and demethoxycurcumin [22].

Clinicals studies further supported that a high single oral dose (up to 12 g/day) of curcuminoids were very well tolerated [14,23].

Several approaches have been used to increase the bioavailability of curcumin including liposomes, polymeric nanoparticles, micelles, extracellular vesicles and other formulations in order to identify drug vehicles; although, they are associated with inherent limitations, such as a short circulation time, as well as stability issues when used as unmodified liposomes in vivo [24,25].

Use of exosomes, as nano drug delivery vehicles, is an emerging area of research and has great potential for the development of novel therapeutic applications [26].

Exosomes are the smaller EVs, which contain different bioactive compounds with their cargo, which could lead to cell behavior changes in the recipient cell. Exosomes as drug carriers have the potential to overcome the limitations associated with other nanoparticle-based technologies [26,27].

There are several advantages of using exosomes as drug carrier systems, such as: low immunogenicity, biodegradability, non-toxicity, a strong cargo-loading and cargo protective capacity, marked ability to overcome natural barriers and penetrate into deep tissues [28], intrinsic cell targeting potential in native structure and deformable conformations [29], and marked ability to cross the blood–brain barrier (BBB) [30,31].

Exosomes contain also microRNA (miRNA), the small noncoding RNAs which act as epigenetic negative/positive regulators in various physiological processes [32,33].

A large number of studies have demonstrated that dietary compounds and bioactive foods could change the expression of various miRNAs involved in various well-known cancer processes such as angiogenesis, cell cycle regulation, apoptosis, differentiation, inflammation, metastasis, and pathways involved in stress response [14,34,35].

miRNAs are one of the important targets for CUR [36]. Several studies indicated that CUR could exert potential anti-cancer properties via targeting miRNAs, such as miRNA-34a, miRNA-21, miRNA-181, miRNA-7, miRNA-9, and miRNA-200c. Moreover, it has been shown that CUR could affect cell sensitivity to chemotherapy via targeting a variety of miRNAs such as miRNA-186, miRNA-21, and miRNA-27a [37,38].

## 3. Curcumin (CUR) and Exosomes

The efficacy of CUR is evidenced by different studies, most of them have involved animal experiments; however, there are several reports about the benefits of curcumin use in humans.

Into the body, the absorption of CUR is poor, and even when absorbed it is rapidly metabolized and excreted [39,40], moreover very high doses (>3.6 g/day in humans) are required to produce possible medicinal effect [41].

An appropriate drug delivery system is necessary for its clinical application, one of these is represented by extracellular vesicles (EVs), that carry a cargo of proteins, lipids, RNA, miRNA, and DNA. Due to their properties of shuttling in-and-out of the cells these particles have been exploited as a possible curcumin carrier [42].

EVs, heterogeneous membranous structures circulating in the extracellular body fluid, have a crucial role in cell–cell signaling representing one of the new emerging modes of cell communication. EVs are involved in many biological responses including inflammation and play a key role in a number of diseases, such as inflammatory bases neurodegenerative diseases and rheumatic diseases [43,44,45,46,47,48,49,50].

EVs are secreted from prokaryotic and a wide variety of eukaryotic cells types, and have been isolated in various body fluids from the main fluids in the organism [51,52].

Classification of the three main types of EVs is based upon performance and size: apoptotic bodies (up to 4000 nm in diameter) and microvesicles (100–1000 nm) are formed by outward budding of the plasma membrane, whereas exosomes are smaller in size (100 nm), are formed and stored within the cell before their release [53], and represent a new focus of research interest.

Among the EVs, exosomes gained a great attention for the delivery of natural compounds. Exosomes contain different bioactive compounds including protein, mRNA, miRNA [54] that, with their cargo, could lead to behavior changes in the cell recipient.

Exosomes could deliver their material to the designated cell recipient via receptor–ligand interaction, direct fusion of membranes, or internalization via endocytosis [55]. After internalization, exosomes may fuse with the limiting membrane of endosomes, resulting in the horizontal genetic transfer of their content to the cytoplasm of target cells. The bioactive molecules contained in exosomes have been shown to impact target cells via the following mechanisms: (1) direct stimulation of target cells via surface-bound ligands; (2) transfer of activated receptors to recipient cells; and (3) epigenetic reprograming of recipient cells via delivery of functional proteins, lipids, and RNAs [56].

Extensive data have shown the use of exosomes as vehicles for therapeutic drug delivery, having desirable features such as a long circulating half-life, intrinsic ability to target tissues, biocompatibility, minimal or no inherent toxicity issue, and are also employed to carry small molecular drugs across the BBB [30,57].

To load exosomes with active compounds, various methods were used, including simple incubation of exosomes and active compounds, sonication of a mixture of exosomes and active compounds, and electroporation of exosomes [31].

There are two major formulations of CUR and exosomes: (a) CUR encapsulated or loaded exosomes (exocur) prepared by loading CUR in the exosome, and (b) CUR-primed exosomes (CUR-EXO) when the cells are treated with CUR and then CUR-EXO are released [58,59,60,61].

Sun et al., for the first time, have shown the use of exosomes as a drug delivery system demonstrating that the anti-inflammatory activity of CUR with an exosomal formulation is remarkably higher when compared to liposomal CUR and free CUR [62].

In 2011, Zhuang et al. delivered CUR-loaded exosomes (ExoCUR) through a nasal route, and studied their effects on inflammatory diseases of the brain, founding a reduction in the number of inflamed microglial cells 2 h after administration, along with an increase of apoptotic events compared to a control group [63].

Kalani et al. administered CUR-loaded embryonic stem cell exosomes (MESC-ExoCUR) through the nasal route in ischemia-reperfusion (IR) injured mice and found that treatment with MESC-ExoCUR improved the stroke volume, ischemia-reperfusion injured neurons, brain vasculature, and vascular junction proteins. More interestingly, it has been shown an improvement in the neurological score after only 3 days of treatment when compared to IR-mice [60].

Emerging evidence has suggested that exosomes released by Human Umbilical Cord Mesenchymal Stem Cells contain miRNAs like let-7b [55].and miR-181c [64] that can specifically bind to the 3’ UTRs of target cellular mRNAs leading to the inhibition of TLR-4 expression and further to the suppression of the downstream NF-κB activity [65].

Aquin and coworkers incubated CUR with milk-derived exosomes and this formulation resulted with increase of 3-5 times in bioavailability of CUR in various organs versus free agent.

ExoCUR showed a significantly higher anti-inflammatory activity measured as NF-κB activation in human lung and breast cancer cells and antiproliferative activity against multiple cancer cell lines including, breast, lung, and cervical cancer [66].

To date, the existing literature does not report articles that consider the exact mechanism by which exosomes-CUR loaded modulate TLR-4 receptor, but surely, they are able to change the behaviour of recipient cell via targeting a sequence of cellular or molecular events associated with cell-signalling pathway.

Therefore, we could speculate that exosomes-CUR loaded may act on TLR-4 receptor by a direct stimulation of the receptor, by regulating target proteins in inflammatory signalling TLR-4 pathway, or by modulation of recipient cells miRNA.

## 4. Toll-Like Receptor 4 (TLR-4) Pathway

### 4.1. Overview about TLR-4 Structure and Expression

Toll-like receptors (TLRs) belong to type I transmembrane proteins and are a panel of conserved pattern-recognition receptors (PRRs) that are activated by a variety of pathogen-associated molecular patterns (PAMPs). TLRs are widely expressed on the surface of a large number of cells including macrophages, monocytes, and dendritic cells. TLRs contain three structural domains: a transmembrane domain, a cytoplasmic Toll/IL-1 receptor (TIR) domain, and a leucine-rich repeats (LRRs) motif. The LRRs motif is responsible for pathogen recognition, whereas the TIR domain interacts with signal transduction adaptors and initiates signaling. This family of receptors is distinctly important for pathogen recognition by the innate immune system [67,68].

They play a vital role in the early innate and adaptive immunity, thus mediating various signaling pathways in sterile and infective inflammation [69,70]. TLRs play a key role in the modulation of the immune responses and stimulate the production of inflammatory chemokines and cytokines, [71] including the transcription factor nuclear factor-κB (NF-κB) signal transduction pathways [72,73].

TLR-4 is one of the most studied TLRs, belongs to the family of PRRs. It is homologue of the *Drosophila* Toll protein [74]. TLR4 is characterized by two domains, an extracellular domain which consists of 608 residues and an intracellular domain which consists of 187 residues. TLR-4 is expressed on the cell surface of both hematopoietic and nonhematopoietic cells, including endothelial cells [75,76,77], cardiac myocytes [78], cells of the CNS [79], thyroid cells [79,80], endometrial cells [81], mesangial cells [82], adipocytes [83], monocytes, dendritic cells, B lymphocytes [84], macrophages [85]. TLR-4 has also been identified in human β-cells and β-cell lines such as HP62 [86] and in the melanoma cell line [87].

Given the leading role of TLR4 in host defense against microbial infections; it specifically recognizes and acts as a receptor for bacterial lipopolysaccharide (LPS) endotoxins, which are biologically important PAMPs. LPS endotoxins are an integrated part of the outer membrane of Gram-negative bacteria. In spite of the structural heterogeneity of LPSs between bacterial species, it is recognized by a cascade of LPS receptors and accessory proteins, LPS binding protein (LBP), CD14, and TLR-4-myeloid differentiation protein 2(MD-2)-complex [88,89,90,91,92].

Multiple transcript variants encoding different isoforms have been found for the gene encoding for this receptor [93]. Mutations in the gene encoding for this receptor have been associated with differences in LPS responsiveness.

### 4.2. TLR-4 General Signal Transduction Pathways

A wide range of available studies in the literature have extensively reviewed TLR-4 signaling pathways, including MyD88-dependent and MyD88-independent pathways [94,95,96,97,98,99]. Four adaptor proteins, involved in these two distinctive intracellular signaling pathways, have been described in the literature: myeloid differentiation primary response protein 88 (MyD88), MyD88-adaptor-like (MAL) protein, also known as TIR domain-containing adaptor protein (TIRAP), TIR domain-containing adaptor inducing IFN-β (TRIF), also known as TIR domain-containing adaptor molecule-1 (TICAM-1), and TRIF-related adaptor molecule (TRAM) [100,101]. In brief, appropriate ligand binding to TLR-4 receptor results in its homodimerization through the interaction between its intracellular TIR domains followed by conformational changes that result in the activation of a downstream cascade of events. TLR-4 recruits a specific combination of adaptors that activate different transcription factors, giving rise to appropriate inflammatory responses. With the exception of TLR-3 and similarly to other TLRs, TLR-4 shares a common adaptor MyD88 to NF-κB and activating protein-1 (AP-1) and induces the expression of various inflammatory cytokines through IL-1R-associated kinase (IRAK), tumor necrosis factor (TNF) receptor-associated factor-6 (TRAF6), and mitogen-activated protein (MAP) kinases (MyD88-dependent pathway) [95].

TLR-4 intracellular signaling is initiated through at least two major pathways: (i) MYD88-TIRAP pathway (also known as MyD88-dependent pathway), where TIRAP mediates the activation of the MyD88-dependent pathway downstream of TLR4 [102,103,104]; it regulates the early activation of NF-κB and triggering the transcription of the related inflammatory cytokines, such as IL-12, IL-8, IL-6, and IL-1 [105,106].

The second TLR-4 pathway is known as (ii) TRIF–TRAM pathway (or MyD88-independent pathway). As described below, this signaling transduction pathway is involved in the induction of type I interferons (IFNs) and Interferons-inducible genes via Interferon regulatory factor-3 activation and other inflammatory mediators.

**Figure 1:**
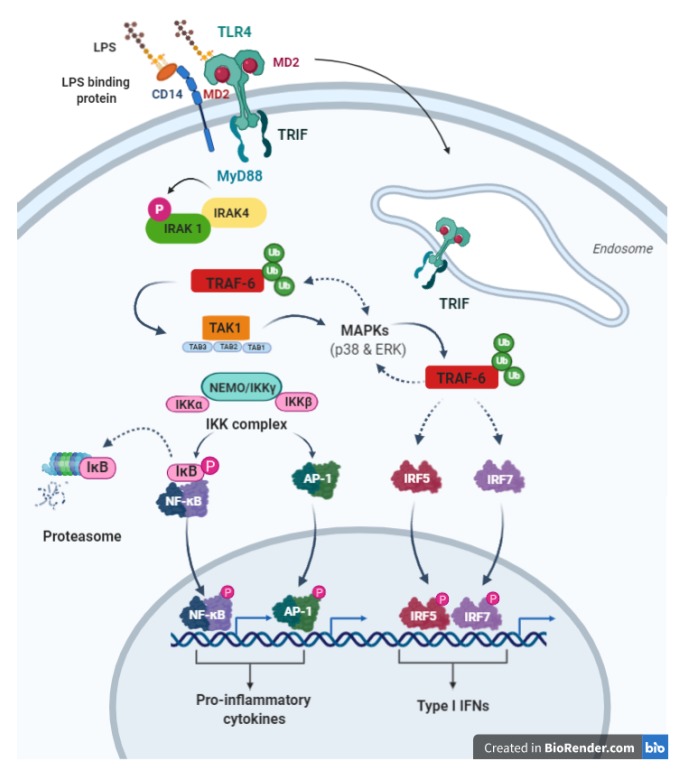
Receptor 4 (TLR4) general signalling transduction pathway. This figure was created with Biorender.com.

The general TLR4 simplified pathway is illustrated in Figure 1.

#### 4.2.1. MYD88-Dependent Pathway (MYD88-TIRAP Pathway)

After TLR activation, MyD88 forms a complex with IRAK-1, also known as Myddosome. During Myddosome formation, IRAK4 catalyzes the phosphorylation of IRAK1 on various sites and induced its activation, then it will be released from MyD88 [107,108,109]. IRAK1 associates with polyubiquitinated TRAF6 and TAK1 protein kinase complex, which belongs to the MAPKKK family and forms complex with TAB1, TAB2 and TAB3 subunits. These subunits interact in turn with polyubiquitin chain generated by TRAF6 to drive the activation of TAK1 [110,111]. TAK1 then activates IKK complex-NF-κB and MAPK through two different pathways. The IKK complex consists of two catalytic subunits IKKα, IKKβ, and the regulatory subunit NEMO (also known as IKKγ). The IKK complex phosphorylates IκBα (NF-κB inhibitory protein), which undergoes proteasome degradation, allowing NF-κB to translocate into the nucleus and induce the expression of the proinflammatory genes. TAK1 binds to the IKK complex through ubiquitin chains, which allows it to phosphorylate and activate IKKβ. TAK1 activates MAPK family members such as JNK ERK1/2, and p38, which in turn mediates the activation of AP-1 family transcription factors or the stabilization of mRNA to regulate inflammatory responses [112].

#### 4.2.2. MYD88-Independent Pathway (TRIF-TRAP Pathway)

The TRIF–TRAM pathway (also known as MyD88-independent pathway), where TRIF is recruited to TLR-4 leading to the activation of the interferon regulatory factor-3 (IRF3) transcription factor, which subsequently up-regulate the levels of expression of certain genes encoding for interferons type I (IFNs) and co-stimulatory factors. Furthermore, TRIF-TRAM dependent pathway enhanced the TNF-α production and regulates its secretion [113,114]. TNF-α, in turn, binds to its specific receptors leading to NF-κB activation. Thus, the TRIF–TRAM pathway is responsible for the activation of the late phase of NF-κB activation via TNF-α and IRF3 secretion and the subsequent production of type I IFN (IFN-α/β), IFN-inducible gene products, and an immune regulatory response [99]. The majority of the LPS-induced responses are MyD88-independent (TRIF-dependent).

## 5. Neuroinflammatory Diseases

Neuroinflammation represents a condition involving the immune response of the nervous system to injury, characterized by the activation of resident glial cells and the recruitment and infiltration of peripheral blood cells into the brain parenchyma [115]. Inflammatory responses in the brain are associated with release of inflammatory mediators, such as cytokines and chemokines, increased levels of prostaglandins (PGs), particularly Prostaglandin E_2_ (PGE2), and generation of oxygen and nitrogen reactive species, that may damage the blood–brain barrier, resulting in further cellular damage and loss of neuronal functions [116].

Neuroinflammation is prevalent in a number of brain diseases, including Alzheimer’s disease (AD), Parkinson’s disease (PD), traumatic brain injury (TBI), amyotrophic lateral sclerosis (ALS), Huntington’s, and many others [117]. Experimental evidence suggests that a strong inflammatory response in the periphery due to lipopolysaccharide (LPS) presence [118] or viral infections [119] may cause subsequent CNS infiltration of peripheral leukocytes, such as T cells and macrophages, which share several functional features with microglia expressing toll-like receptors (TLRs) and class II major histocompatibility complex and eliciting the ability to present antigens to CD4+ T lymphocytes [118].

Glial cells are essential players in CNS: they orchestrate both CNS development and homeostasis and modulate neuronal communication other that participate in CNS degeneration/regeneration during disease or injury [120]. Among glial cells, microglia and astrocytes are critical in assuring optimal milieu for neuronal function. Astrocytes maintain CNS homeostasis and promote neuronal survival by regulating metabolites traffic and blood flow [121,122,123]. In the course of CNS injury and disease, they exhibit an activated state, in a process called reactive astrogliosis, contributing to both inflammation and reparative processes [124].

In the CNS, microglia activation plays a central role in the pathophysiology of neurodegenerative disease, since these cells act as sensors for perturbed brain tissue homeostasis, and function as professional CNS phagocytes. Microglial activation is routinely divided into two main phenotypes, M1 (classical activation) and M2 (alternative activation), although the existence of different microglia subtypes under disease conditions is described [125]. M1 cells are activated by LPS/IFN-γ and upregulate pro-inflammatory mediators, including IL-1β, IL-6, ROS, iNOS, and TNF-α. M2 microglia is achieved upon treatment with IL-4/10/13 or TGF-β, and triggers upregulation of anti-inflammatory genes including arginase-1, mannose receptor (CD206), YM1, and FIZZ [126,127].

Microglial cells represent the main source of reactive oxygen species and nitrogen species, TNF-α, and glutamate, all of which are neurotoxic when released at a high dose by TLR-4 triggered microglia [128], as observed in the case of AD, MS, PD, and ALS patients [129,130,131].

However, additional studies have allowed scientists and clinicians to have a better understanding of the benefit derived from the multifunctionality of microglia, since activated microglia is not always harmful being also able to produce anti-inflammatory mediators and neurotrophic factors such as insulin-like growth factor-1, glial cell-derived neurotrophic factor, brain-derived neurotrophic factors, and other factors [132,133,134] leading to beneficial effects.

Therefore, differential activation of microglia cells represents the central point that regulates neuroinflammation resulting in neurotoxicity or neuroprotection. Consequently, the microenvironment in which the neurons are located present the crucial element capable of marking the fate of neurons in terms of survival or degeneration.

## 6. CUR and TLR-4 in Neuroinflammatory Diseases

Microglia express diverse receptors, including TLR-4, the major LPS receptor [91,135]. The binding of LPS to TLR4–MD-2 complex induces the production of pro-inflammatory molecules, such as TNF-α, IL-1β, and IL-6, chemokines, enzymes, and reactive oxygen and nitrogen species by microglia cells [136]. Thus, targeting microglia and TLR4–MD-2 complex activation is gaining increasing interest as a potential therapeutic or preventive strategy for the treatment of CNS disorders.

CUR being able to cross the BBB maintaining its biological activity [137,138] is studied as possible therapy for neuroinflammatory and neurodegenerative diseases. Different studies have demonstrated that CUR attenuates inflammatory response via TLR-4 pathway.

In 1999, Jobin et al. found that CUR has powerful anti-inflammatory effects because it suppressed the activation of NF-κB induced by various pro-inflammatory stimuli, through inhibition of IKKβ kinase activity or DNA binding of p65 [139,140]. Interesting studies analyzed more deeply this mechanism showing that curcumin contains α, β-unsaturated carbonyl group, and this group inhibits TLR-4 activation by interfering with receptor dimerization [140] an important step for the activation of downstream signaling pathways of this receptor [141,142]. According to Youn et al., the target of curcumin is TLR-4, but not the downstream components of TRIF pathway [141]. Indeed, CUR did not inhibit IRF3 activation induced by another immediate TLR-4 downstream component. These results suggested that dimerization of TLR-4 receptor can be a target for phytochemicals and pharmacological agents to ameliorate chronic inflammatory diseases.

In addition, Zhu et al. in 2014 have shown that administration of CUR may improve neuroinflammatory process by reducing microglia/macrophage activation and neuronal apoptosis through a mechanism involving the TLR4/MyD88/NF-κB signaling pathway in microglia/macrophages [143].

Also, Gao et al. in a recent study have found that during subarachnoid hemorrhage, CUR could reduce neuroinflammatory response, via shifting microglia phenotype toward M2, by inhibition of TLR4/MyD88/NF-κB signaling pathway [144].

The anti-inflammatory activity of CUR and its derivatives with an α, β-unsaturated 1,3-diketone moiety is due to their ability to coordinate Mg2+, affecting the proper assembly of the TLR4-MD-2-LPS ternary complex [143].

Recent studies have shown that Triggering Receptor Expressed on Myeloid cells 2, also known as (TREM2), is an efficient negative regulator of TLR-4 signaling [145,146,147]. TREM2 is a membrane receptor that mediates critical functions of microglia, such as suppression of pro-inflammatory cytokines and promotion of phagocytosis of apoptotic neurons and cell debris [148]. If TREM2 expression is reduced in macrophages or dendritic cells, inflammatory cytokine production after TLR activation is enhanced [148,149]. In vivo studies suggested that TREM2 modulated neuroinflammatory responses involving a negative regulatory mechanism of the TLR4-mediated activation of NF-κB signaling pathways [150]. Zhang et al. have found the negative correlation between TLR-4 and TREM2 after LPS stimulation of BV2 cells, CUR was able to reverse LPS-induced hyperactivity of TLR-4 and NF-κB by a markedly increasing TREM2 expression in BV2 cells. CUR therefore facilitates microglial polarization from the pro-inflammatory phenotype towards the anti-inflammatory also inhibits LPS-induced neuroinflammatory response by reducing the imbalance of TREM2 and TLR-4-mediated NF-κB activation. This study has provided a deeper understanding of curcumin’s therapeutic potential in the treatment of microglia-mediated neuroinflammatory diseases [151].

The positive effects of CUR are not limited to improve neuroinflammatory diseases by modulation of TLR-4 in LPS stimulated cells

Zhu et al. have found that 24 h following a traumatic brain injury (TBI), TLR4 protein expression in pericontusional tissue reached a maximal level. Administration of CUR after injury, can improve patient outcomes by decreasing the acute activation of microglia/macrophages and neuronal apoptosis via a mechanism involving TLR-4/MyD88/NF-ĸB signaling pathway [152].

In addition, Yu et al. demonstrated that the administration of CUR prevents the inflammatory and oxidative mediators in 1-methyl-4-phenylpyridinium ion-(MPP+-)-induced mesencephalic astrocytes. The action of curcumin results in an inhibition of TLR-4 and its downstream signaling pathway including NF-κB, IRF3, MyD88, and TIRF that are induced by MPP++-stimulated astrocytes. This leads to the conclusion that CUR administration could be a potential agent that exhibits anti-inflammatory properties against inflammation implicated in the pathogenesis of PD [143].

Recent reports suggest that due to curcumin’s ability to reduce Beta-amyloid plaques, delay degradation of neurons, as well as decrease inflammatory microglia responses, the overall memory in patients with AD results significantly improved [153,154].

New findings attribute to CUR a neuroprotective potential also in ethanol-associated neurodegenerative diseases by regulation of ROS, TLR-4, and receptor for advanced glycation [155].

In conclusion, we could assert that in neuroinflammatory diseases, TLR-4 receptor is modulated by curcumin that exerts a neuroprotective effect acting directly on receptor or on its downstream pathway. This can be further exploited for designing future therapeutic strategies against neuroinflammatory diseases.

## 7. Rheumatic Diseases

Rheumatic diseases include several pathological conditions which can involve many tissues and organs but mainly affect joints and musculoskeletal system. Owing to the large heterogeneity of physiopathological processes and clinical manifestations, the classification and description of epidemiology of these disorders can be challenging. Rheumatic diseases include pathological conditions affecting joint and periarticular tissues, muscles, connective tissues including bone, and internal organs. They represent the most frequent cause of morbidity and disability, especially in western countries, and can significantly decrease the quality of life of affected people, inducing a relevant economic burden for healthcare services [156].

Depending on the prevailing etiology and pathophysiological mechanisms, rheumatic diseases can be classified into inflammatory/autoimmune and degenerative disorders.

The most frequent inflammatory rheumatic diseases comprise Rheumatoid Arthritis (RA) and Spondiloartritis (SpA). RA is an inflammatory autoimmune disease, predominantly characterized by chronic inflammation of synovial joints which can lead to severe joint damage, and by extra-articular manifestations that include lung involvement, vasculitis, rheumatoid nodules, and systemic comorbidities [157]. The term SpA indicates a group of inflammatory diseases sharing similar features, affecting the axial and peripheral joints and the enthuses, the sites where ligaments and tendons connect to bone, and includes ankylosing spondylitis, psoriatic arthritis, reactive arthritis, enteropathic arthritis, and undifferentiated SpA [158].

On the other hand, the major degenerative/metabolic rheumatic diseases are represented by Osteoarthritis (OA) and Osteoporosis (OP). OA is the most diffuse chronic joint disease, characterized by the progressive degradation and loss of joint cartilage and changes occurring also in other joint components, such as subchondral bone and synovial membrane [159,160]. According to World Health Organization, OP is a systemic skeletal disorder characterized by low bone mass and micro-architectural deterioration of bone tissue, leading to enhanced bone fragility and a consequent increase in fracture risk [161]. The less frequently occurring systemic autoimmune diseases are connective tissue diseases such as systemic lupus erythematosus, systemic sclerosis, Sjogren’s syndrome, polymyositis, and dermatomyositis, which can involve not only the musculoskeletal system and skin but also other organs and tissues, including lung, kidney, gastrointestinal tract, heart, and blood vessels. These disorders are characterized by very heterogeneous clinical manifestations ranging from mild symptoms to potentially life-threatening visceral involvement [162].

Musculoskeletal pain represents the main symptom of these diseases, many of which have a chronic and progressive course, often characterized by periods of both remission and exacerbation. Treatment of inflammatory/autoimmune rheumatic diseases includes conventional and biologic disease-modifying anti-rheumatic drugs, immunosuppressant drugs and corticosteroids [163], whereas the first line treatment to control pain in both degenerative and inflammatory joint diseases is represented mainly by non-steroidal anti-inflammatory drugs [164]. Some clinical and experimental data provide evidences that curcumin presents anti-oxidative capacity and it is able to decrease inflammation trough an inhibitory effect on cyclooxygenase and NF-κB pathways, as these pathways are involved in the pathogenesis of rheumatic diseases, it is rational to support the hypothesis that curcumin can have a possible therapeutic role in the treatment of symptoms of these disorders.

## 8. CUR and TLR-4 in Rheumatic Diseases Inflammatory Arthritic Diseases

The renowned multi-target anti-inflammatory properties of curcumin [165] have led to an increased interest in its potential therapeutic role in rheumatic diseases over recent years.

Many studies focused on OA, a degenerative joint disease mainly affecting elderly, characterized by cartilage degradation, subchondral bone sclerosis, osteophyte formation, synovial inflammation, and angiogenesis of affected tissues. The periarticular structures may also be involved, such as muscles, nerves, and bursa. Therefore, OA affects the entire joint structure and leads to pain, deformity, and loss of function [166]. Several clinical trials suggest the beneficial role of curcumin in OA patients, showing improvement in clinical outcomes like walking distance, the Western Ontario and McMaster Universities Arthritis Index, and a decrease in inflammatory markers expression, such as IL-1β levels, erythrocyte sedimentation rate, IL-6, C-reactive protein (CRP), or oxidative stress markers after curcumin oral supplementation. Furthermore, curcumin showed similar efficacy to non-steroidal anti-inflammatory drugs in the treatment of OA, with decreased side effects (particularly gastrointestinal adverse effects) [167].

A recent study conducted by Yan et al. [168] suggested that the aforementioned beneficial role of curcumin administration in OA may be related to the inhibition of TLR-4 signal. Indeed, TLR-4 plays an important role in the pathogenesis of OA [169]. Several damage-associated molecular patterns (DAMPs) generated from cartilage damage can activate TLR-4 [169]. Moreover, the expression of TLR-4 in joint tissues increases with increasing severity of OA; this leads to inflammatory and catabolic responses by chondrocytes, represented by enhanced expression of inflammatory cytokines such as IL-1, IL-6, TNFα, and matrix metalloproteinase (MMP), release of nitric oxide (NO), and prostaglandin E2 (PGE2) synthesis, which are mediated by NF-κB [168,169,170]. TRL-4 is also involved in the age-related and mechanical stress-related OA, as the accumulation of advanced glycation end products, typical of aging, enhances the expression of catabolic factors by chondrocytes via TLR-4 [171] and shear stress induces a rapid and transient expression of TLR-4 by chondrocytes [172].

Yan et al. showed the relation between intra-articular administration of CUR and TLR-4 inhibition in a rat OA model surgically induced by anterior cruciate ligament transection [168]. The injection of LPS into rat osteoarthritic knees led to an expected increased cartilage damage, higher percentage of apoptotic chondrocytes and amplified synovial inflammation, with higher synovial fluid concentrations of IL-1β and TNFα compared to non-LPS injected rat OA models. However, these effects were reversed after local curcumin administration that attenuated the LPS-induced overexpression of TLR-4 and its downstream NF-κB. The intra-articular CUR injection significantly ameliorated the histological lesion and matrix degradation in articular cartilage, reversed the chondrocyte apoptosis, decreased the IL-1β and TNF-α concentrations in synovial lavage. These data suggest that the anti-inflammatory role of curcumin in OA is strictly connected to the TLR-4 pathway [168].

The pathogenic role of TLR-4 and the potential therapeutic effect of CUR have been evaluated also in gout, a common inflammatory arthritis due to the deposition of monosodium urate (MSU) crystals in articular and periarticular tissues [173]. The TLR-4/NF-κB/IL-1 pathway plays a crucial role in the development of acute inflammation in gout patients [174], and this signalling pathway seems to be the therapeutic target of CUR in a recent study conducted by Chen et al. [175]. In this study, authors investigated the effects of CUR administration in MSU crystal-induced inflammation models in vitro and in vivo. As expected, they have found high levels of TLR-4 and MyD88- in MSU-stimulated THP-1 cells; after CUR treatment, these levels were significantly reduced. Moreover, in mouse models of gout arthritis induced by intra-articular injection of MSU, CUR treatment led to improved clinical conditions represented by reduced joint swelling, and inhibited the expression of TLR-4 and MyD88 [175].

Additional research is needed in other rheumatic diseases, for instance rheumatoid arthritis (RA) and systemic lupus erythematosus. More interestingly, Available data suggest a potential role played by CUR in therapeutic strategy for these diseases, showing decreased disease activity and CRP levels in RA patients and improved laboratory outcome in lupus nephritis after CUR administration [165].

## 9. Conclusions

Several studies have reported the efficacy of phytochemicals as anti-inflammatory compounds, and curcumin represents one of the top scientific interest in the last year, with its wide range of therapeutic activities.

It has been shown that CUR has anti-inflammatory property modulating TLR-4 receptor and its downstream pathway.

CUR bioavailability is very low in humans, so an appropriate drug delivery system is necessary for its clinical application, exosomes CUR-loaded could improve the efficacy of CUR even if the exact mechanism of action is still unknown.

Further explorations have been done to increase bioavailability of CUR, in this direction exosomes CUR-loaded could represent one of the new emerging areas of research to improve the prognosis of neuro-inflammatory and rheumatic diseases.

## Abbreviations

CUR	Curcumin
TLR4	The toll-like receptor 4
EVs	extracellular vesicles
exocur	curcumin encapsulated or loaded exosomes
CUR-EXO	curcumin-primed exosomes
MESC-ExoCUR	curcumin-loaded embryonic stem cell exosomesEsosomes Curcumin
miRNA,	miRNAs, MicroRNAs
TLRs	Toll-like Receptors
PRRs	pattern-recognition receptors
PAMPs	pathogen-associated molecular patterns
TIR	Toll/IL-1 receptor; LRRs, leucine-rich repeats
NF-κB	nuclear factor-κB; LPS, lipopolysaccharide
LBP	LPS binding protein
MD-2	myeloid differentiation protein 2
MyD88	myeloid differentiation primary response protein 88
MAL	MyD88-adaptor-like
TIRAP	TIR domain-containing adaptor protein
TRIF	TIR domain-containing adaptor
TICAM-1	TIR domain-containing adaptor molecule-1
TRAM	TRIF-related adaptor molecule
AP-1	activating protein-1
IRAK	IL-1R-associated kinase
TNF	tumour necrosis factor
TRAF6	receptor-associated factor-6
MAP	mitogen-activated protein
IRAK-1	kinase Interleukin-1 receptor-associated kinase
TRAF6	TNF Receptor Associated Factor 6
IRF3	interferon regulatory factor-3
CNS	central nervous system
TLRs	toll-like receptors
TNF	tumor necrosis factor
IL	interleukin
IRF3	interferon regulatory factor 3
TREM2	Triggering Receptor Expressed on Myeloid cells 2
TBI	traumatic brain injury
RA	Rheumatoid Arthritis
SpA	Spondiloartritis
OA	osteoarthritis
CRP	C-reactive protein
DAMPs	damage-associated molecular patterns
MMP	matrix metalloproteinase
NO	nitric oxide
PGE2	prostaglandin E2
AGEs	advanced glycation end products
MSU	monosodium urate
RA	rheumatoid arthritis
